# circTLK1 facilitates the proliferation and metastasis of renal cell carcinoma by regulating miR-495-3p/CBL axis

**DOI:** 10.1515/biol-2021-0041

**Published:** 2021-04-15

**Authors:** Xiangli Lei, Meiling Yang, Zhifang Xiao, Heng Zhang, Shuai Tan

**Affiliations:** Department of Nephrology, Affiliated Nanhua Hospital, University of South China, Hengyang, Hunan, China; Department of Oncology, Affiliated Nanhua Hospital, University of South China, 336 Dongfeng Road, Zhuhui District, Hengyang, 421000, Hunan, China; Department of Endocrinology, Affiliated Nanhua Hospital, University of South China, Hengyang, Hunan, China; Department of Hematology, Affiliated Nanhua Hospital, University of South China, Hengyang, Hunan, China

**Keywords:** circTLK1, miR-495-3p, CBL, renal cell carcinoma

## Abstract

Renal cell carcinoma (RCC) is a common urological malignancy. Circular RNAs (circRNAs) have been confirmed to play an important regulatory role in various cancers. This study aimed to investigate the role and potential mechanism of circTLK1 (hsa_circ_0004442) in RCC. The levels of circTLK1, Cbl proto-oncogene (CBL), and microRNA-495-3p (miR-495-3p) were detected by quantitative reverse transcription polymerase chain reaction or western blot. Cell proliferation, cycle arrest and apoptosis, migration, and invasion were assessed by colony formation, flow cytometry, scratch, and transwell assays. The levels of E-cadherin and Vimentin were measured by western blot. The targeting relationship between miR-495-3p and miR-495-3p or CBL was verified by dual-luciferase reporter assay. Tumor growth *in vivo* was evaluated by xenograft assay. The results found that circTLK1 and CBL were up-regulated in RCC tissues and cells. Silencing of circTLK1 or CBL inhibited proliferation and metastasis and accelerated apoptosis in RCC cells. In addition, circTLK1 directly bound to miR-495-3p, and CBL was the target of miR-495-3p. circTLK1 sponged miR-495-3p to increase CBL expression. Moreover, knockdown of circTLK1 suppressed tumor growth *in vivo*. In conclusion, down-regulation of circTLK1 restrained proliferation and metastasis and promoted apoptosis in RCC cells by modulating miR-495-3p/CBL axis.

## 1 Introduction

Renal cell carcinoma (RCC) is a urologic malignancy originating from the renal epithelium, which accounts for more than 90% of renal cancers [1]. It is estimated that there were 403,262 new kidney cancer cases and 175,098 related deaths worldwide in 2018 [2]. RCC is a genitourinary malignant tumor with a mortality rate second only to bladder cancer [3]. Targeted therapies have become the main treatment for patients with recurrent or metastatic RCC [4]. Because of the radioresistance and chemoresistance of RCC, the 5-year survival rate of metastatic RCC is still as low as about 10% [5]. Therefore, exploring the potential mechanism of RCC pathogenesis is essential for the development of effective RCC treatment strategies.

Circular RNAs (circRNAs) are a special type of transcripts characterized by covalent closed loops with no 5′ to 3′ polarity [6]. Increasing evidence has manifested that circRNAs exert crucial effects in various diseases, especially cancer, and may be diagnostic or prognostic markers as they are more stable than linear RNA [7]. Moreover, substantial studies have corroborated that circRNAs participate in the occurrence and development of various cancers through mediating diverse biological processes [8]. For example, circ_0000190 hindered cell proliferation and metastasis in gastric carcinoma by down-regulating microRNA-1252 to up-regulate PAK3 [9]. In addition, hsa_circ_0101145 contributed to epithelial-mesenchymal transition (EMT) in hepatocellular carcinoma through absorbing microRNA-548c-3p to regulate laminin subunit gamma 2 expression [10]. In non-small cell lung carcinoma, circ_0000376 facilitated tumor progression and elevated chemoresistance via combining with microRNA-384 [11]. Moreover, a recent study suggested that hsa_circ_0004442 derived from Tousled-like kinases 1 (TLK1) was remarkably up-regulated in RCC, and circTLK1 expedited RCC proliferation and metastasis by modulating microRNA-136-5p/CBX4 pathway [12]. Nevertheless, the exact mechanism of circTLK1 in RCC development still needs further exploration.

Mounting evidence has verified that microRNAs (miRNAs) suppress mRNA translation or induce mRNA degradation by directly pairing with mRNA 3′UTR [13]. Moreover, circRNAs participate in the post-transcriptional regulation of mRNAs by competitively binding to miRNAs [14]. Therefore, we predicted some miRNAs that might bind to circTLK1 through bioinformatics analysis.

Cbl proto-oncogene (CBL) belongs to the E3 ubiquitin ligase family and regulates signal transduction through tyrosine kinase-dependent pathways [15]. Decreasing c-Cbl activity contributes to osteoblast differentiation in mesenchymal-derived osteoblasts [16]. In addition, Cbl-b is an important regulator of innate and adaptive immunity, thus playing a critical role in immune-mediated diseases [17]. However, the role of CBL and circRNA has not been studied.

Herein, we studied the expression and function of circTLK1 and CBL in RCC. Furthermore, we investigated the potential mechanism of circTLK1 in the progression of RCC. These findings might provide new biomarkers for RCC therapy.

## 2 Materials and methods

### 2.1 Clinical samples

RCC tissues (*n* = 35) and adjacent normal tissues (*n* = 35) were obtained from RCC patients undergoing surgery at Affiliated Nanhua Hospital, University of South China between January 2015 and December 2017. Some clinicopathological parameters of RCC patients are listed in Table 1.

**Table 1:** Correlation between circTLK1 expression and clinicopathological parameters in RCC patients

Clinicopathological factors	Number	circTLK1 expression		*P*-value
		Low (*n* = 17)	High (*n* = 18)	
**Age**				
<50 years	19	9	10	>0.05
≥50 years	16	8	8	
**Gender**				
Female	21	10	11	>0.05
Male	14	7	7	
**Tumor size**				
>4 cm	17	5	12	<0.05
≤4 cm	18	12	6	
**Lymph node metastasis**				
Negative	16	11	5	<0.05
Positive	19	6	13	
**TNM stage**				
I–II	15	11	4	<0.05
III–IV	20	6	14	


**Informed consent:** Informed consent has been obtained from all individuals included in this study.
**Ethical approval:** The research related to human use has been complied with all the relevant national regulations and institutional policies and in accordance with the tenets of the Helsinki Declaration, and has been approved by the Ethics Committee of Affiliated Nanhua Hospital, University of South China.

### 2.2 Cell culture

Human normal kidney cell line (HK-2) and RCC cell lines (Caki-1 and 786-O) were commercially acquired from American Type Culture Collection (ATCC, Manassas, VA, USA). All cells were cultured in RPMI-1640 medium (Gibco, Los Angeles, CA, USA) supplemented with 10% fetal bovine serum (FBS; Gibco). All cells were maintained in an incubator with 5% CO_2_ at 37°C.

### 2.3 Cell transfection

Small interfering RNA against circTLK1 (si-circTLK1) or CBL (si-CBL) and negative control (si-NC), CBL overexpression vector (pcDNA-CBL) and the empty vector pcDNA3.1 (pcDNA-NC), miR-495-3p mimic and the control (miR-NC mimic), circTLK1 overexpression vector (oe-circTLK1) and the empty vector pCD5-ciR (oe-NC), and miR-495-3p inhibitor and the control (miR-NC inhibitor) were synthesized by Genechem (Shanghai, China). Lipofectamine 3000 (Invitrogen, Carlsbad, CA, USA) was used for cell transfection when cell confluence reached ∼80%.

### 2.4 Quantitative reverse transcription polymerase chain reaction (qRT-PCR)

RNA was isolated from tissues and cells using Trizol reagent (Solarbio, Beijing, China). For RNase R digestion analysis, RNA (2 μg) was treated with or without RNase R (Seebio, Shanghai, China) for 30 min. Subsequently, the complementary DNA (cDNA) was synthesized using specific reverse transcription kits (Takara, Dalian, China). Then, RNA levels were detected using SYBR Premix Ex Taq (Takara) and calculated using the 2^−ΔΔCt^ method. The PCR amplification procedure included 95°C for 10 min, followed by 40 cycles of 95°C for 5 s, 60°C for 10 s, and 72°C for 10 s. β-Actin or U6 was considered as an internal control. The primers are presented in Table 2.

**Table 2:** The primer sequences for qRT-PCR

Primer name	Sequence (5′–3′)	*T*_m_ (°C)
circTLK1-F	CAGTCAATGGAGCAGAGAA	60.0
circTLK1-R	CCATTCTTGTTGCCTTTTTG	59.1
TLK1-F	ACGTGGCCACAAAATTAGCG	64.7
TLK1-R	GGAGAAGGGCTATTCGGTCG	65.0
CBL-F	TGACATCTTTACCCGACTC	59.4
CBL-R	CATACCCAATAGCCCAC	57.1
miR-495-3p-F	AACACGCAAACAAACATGGTGC	74.5
miR-495-3p-R	CAGTGCAGGGTCCGAGGT	61.1
β-Actin-F	GTCACCGGAGTCCATCACGAT	66.8
β-Actin-R	TCACCAACTGGGACGACATG	65.1
U6-F	CTCGCTTCGGCAGCACA	65.6
U6-R	AACGCTTCACGAATTTGCGT	64.5

### 2.5 Colony formation assay

After transfection, Caki-1 and 786-O cells were trypsinized and then seeded into 6-well plates. The culture medium was changed every 3 days for 2 weeks. Subsequently, the cells were fixed with formaldehyde and stained with crystal violet (Solarbio). Finally, the number of colonies was counted in five randomly selected fields under a microscope.

### 2.6 Flow cytometry

The transfected Caki-1 and 786-O cells were harvested and trypsinized. Subsequently, the precipitate was washed in phosphate-buffered saline (PBS; Solarbio) and fixed with ethanol for 1 h. After incubation with RNase (Seebio) for 30 min, the cells were stained with propidium iodide (PI; Abcam, Cambridge, UK). Finally, cell distribution was monitored by FACScan Flow Cytometry (BD Biosciences, San Diego, CA, USA).

Cell apoptosis was assessed using Annexin V-FITC/PI Apoptosis Detection kit (Vazyme, Nanjing, China) following the manufacturer’s instructions. The apoptosis cells were measured by FACScan Flow Cytometry (BD Biosciences).

### 2.7 Scratch assay

The transfected Caki-1 and 786-O cells were plated in 6-well plates. Later, a linear wound was created by scraping the cells with a sterilized pipette tip. After 24 h of incubation, the migration distance was photographed using a microscope at 100× magnification and calculated using ImageJ 1.8.0 software (National Institutes of Health, Bethesda, MD, USA).

### 2.8 Transwell assay

Cell migration and invasion were determined using transwell chambers with 8 μm polycarbonate membrane filters (Corning, Corning, NY, USA). The transfected Caki-1 and 786-O cells were injected into the upper chamber. Meanwhile, medium with 10% FBS was added as an attractant in the lower chamber. After 24 h of culture, the cells were fixed with methanol and stained with 0.1% crystal violet (Solarbio). The migrated cells were counted under a microscope at 100× magnification. For cell invasion test, the difference was that transwell chamber was pre-coated with Matrigel (Corning).

### 2.9 Western blot assay

Total protein was extracted with RIPA buffer (Solarbio). After the protein was quantified using BCA™ Protein Assay Kit (Pierce, Appleton, WI, USA), the equal amounts of protein samples were separated by polyacrylamide gel electrophoresis and transferred to polyvinylidene fluoride membranes (Millipore, Billerica, MA, USA). The membranes were blocked with 5% fat-free milk for 2 h and incubated with primary antibodies against E-cadherin (1:500, ab15148, Abcam), Vimentin (1:2,000, ab137321, Abcam), CBL (1:5,000, ab32027, Abcam), or β-actin (1:2,000, ab8227, Abcam). After washing thrice with tris-buffered saline, the membranes were probed with horseradish peroxidase-labeled secondary antibody (1:25,000, ab205718, Abcam). Finally, the protein bands were measured using the ECL system (Beyotime, Shanghai, China).

### 2.10 Dual-luciferase reporter assay

circTLK1 sequence containing miR-495-3p wild-type or mutant binding site was cloned into pmirGLO vector (LMAI Bio, Shanghai, China) to form WT-circTLK1 and MUT-circTLK1 vectors. Meanwhile, CBL 3′UTR harboring miR-495-3p wild-type or mutant binding site was inserted into pmirGLO vector (LMAI Bio) to form WT-CBL 3′UTR and MUT-CBL 3′UTR vectors. Next, the constructed vector and miR-NC mimic or miR-495-3p mimic was co-transfected into Caki-1 and 786-O cells. Subsequently, the luciferase intensity was detected via Dual-Lucy Assay Kit (Solarbio).

### 2.11 Xenograft assay

Five-week-old BALB/c nude mice (*n* = 10) were randomly divided into two groups (*n* = 5 in each group). Lentivirus containing circTLK1 short hairpin RNA (sh-circTLK1) or negative control (sh-NC) was purchased from Genechem. 786-O cells (5 × 10^6^) stably expressing sh-circTLK1 or sh-NC were subcutaneously injected into the back of mice. Tumor volume was measured once a week. After 4 weeks, the mice were killed and the xenograft tumors were weighed. The levels of circTLK1, miR-495-3p, and CBL in the excised tumors were measured by qRT-PCR or western blot.


**Ethical approval:** The research related to animal use has been complied with all the relevant national regulations and institutional policies for the care and use of animals and has been approved by the Animal Ethics Committee of Affiliated Nanhua Hospital, University of South China.

### 2.12 Statistical analysis

All data were expressed as mean ± standard deviation using GraphPad Prism 7 software (GraphPad, San Diego, CA, USA). Student’s *t*-test and one-way analysis of variance were used to analyze the differences. The linear relationships among circTLK1, miR-495-3p, and CBL were tested by Spearman’s correlation coefficient. *P* < 0.05 was considered statistically significant.

## 3 Results

### 3.1 circTLK1 and CBL are up-regulated in RCC tissues and cells

**Figure 1:**
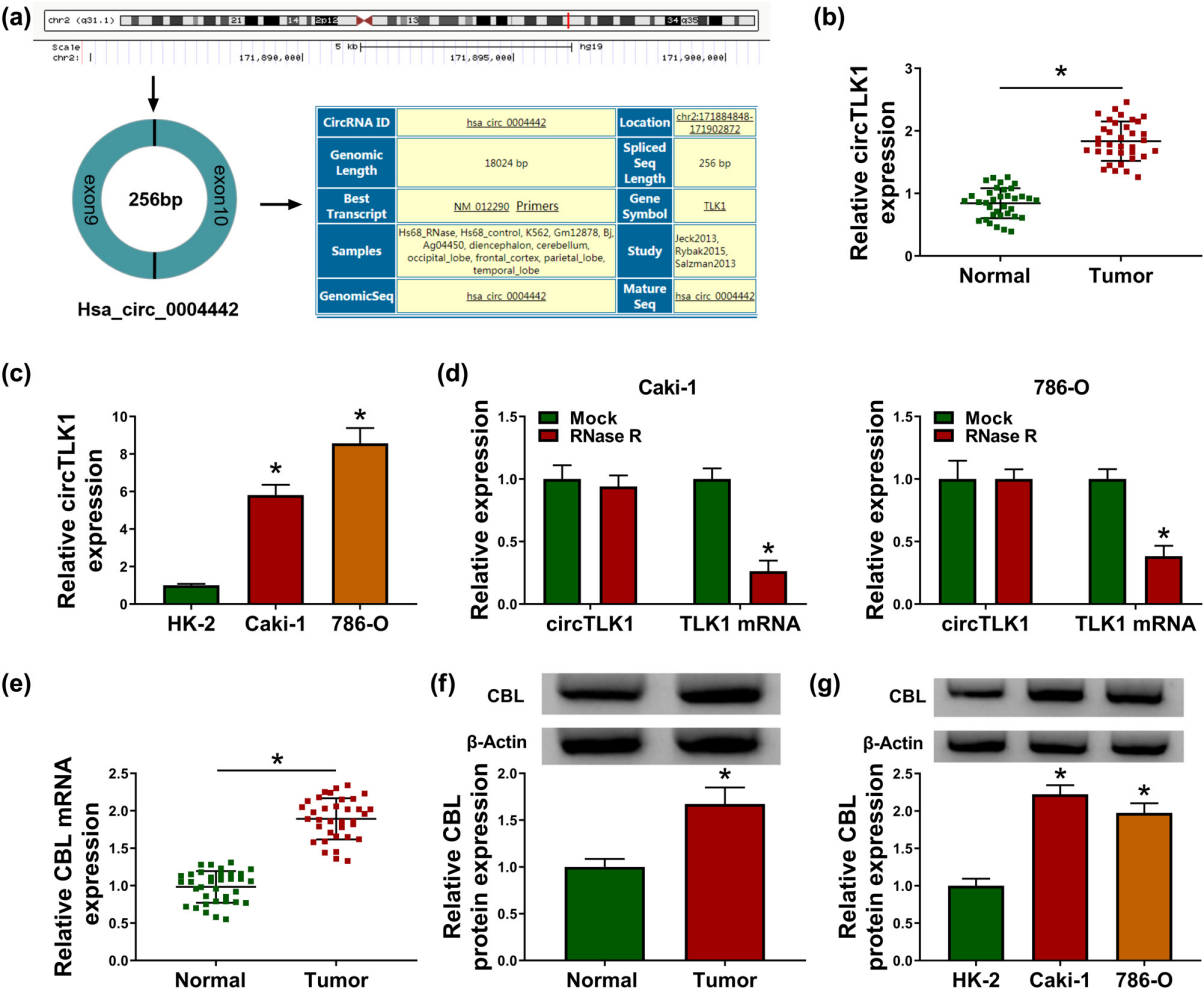
circTLK1 and CBL are up-regulated in RCC tissues and cells. (a) Schematic diagram showing the origin and formation of circTLK1. (b) circTLK1 expression was detected in RCC tissues (*n* = 35) and adjacent normal tissues (*n* = 35) by qRT-PCR. (c) circTLK1 level was measured in HK-2 cells and RCC cells (Caki-1 and 786-O). (d) After RNase R stimulation, the levels of circTLK1 and TLK1 mRNA were examined using qRT-PCR. (e and f) The mRNA and protein levels of CBL in RCC tissues and normal tissues were detected by qRT-PCR and western blot. (g) CBL protein level was measured in HK-2, Caki-1 and 786-O cells. *P < 0.05.

First, we demonstrated that hsa_circ_0004442 was derived from exons 9 and 10 of TLK1 gene (Figure 1a). To explore the biological function of circTLK1 in RCC, the expression differences of circTLK1 in 35 pairs of RCC tissues and normal tissues were analyzed. As shown in Figure 1b, circTLK1 expression was remarkably higher than that in normal tissues. Meanwhile, circTLK1 level was strikingly increased in RCC cells (Caki-1 and 786-O) compared with normal human kidney cell line (HK-2) (Figure 1c). Moreover, RNase R digestion assay showed that circTLK1 was not affected by RNase R, indicating that circTLK1 was more stable than TLK1 mRNA in Caki-1 and 786-O cells (Figure 1d). In addition, CBL mRNA and protein levels in RCC tissues were markedly higher than those in normal tissues (Figure 1e and f). Simultaneously, CBL protein expression in Caki-1 and 786-O cells was significantly increased compared to HK-2 cells (Figure 1g). As presented in Table 1, circTLK1 expression was not associated with age and gender, but was associated with tumor size, lymph node metastasis, and TNM stage. These data hinted that circTLK1 and CBL might play carcinogenic roles in RCC.

### 3.2 Knockdown of circTLK1 inhibits proliferation and metastasis and promotes apoptosis in RCC cells

**Figure 2:**
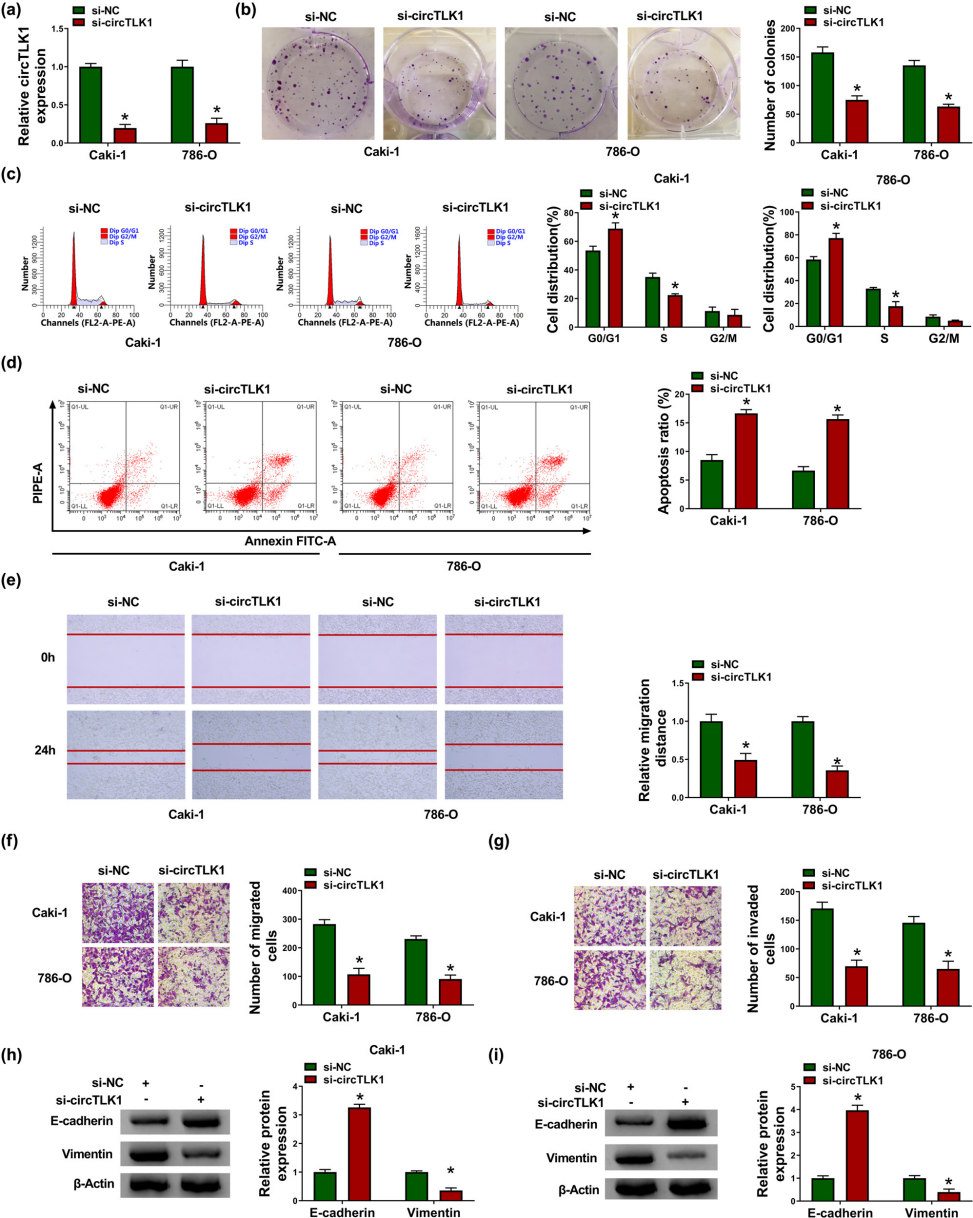
Knockdown of circTLK1 inhibits proliferation and metastasis and promotes apoptosis in RCC cells. (a) The knockdown efficiency of circTLK1 was determined by qRT-PCR. After transfecting Caki-1 and 786-O cells with si-NC or si-circTLK1, colony formation assay and flow cytometry were used to evaluate colony number (b), cell distribution (c), and apoptosis ratio (d). (e–g) Cell migration and invasion were assessed by scratch assay and transwell assay in Caki-1 and 786-O cells transfected with si-NC or si-circTLK1. (h and i) The levels of EMT-related proteins (E-cadherin and Vimentin) were detected using western blot. *P < 0.05.

To investigate the function of circTLK1 in RCC development, we performed loss-of-function experiments by transfecting si-circTLK1 into Caki-1 and 786-O cells. First, circTLK1 level in the si-circTLK1 group was prominently reduced compared with the si-NC group, suggesting that circTLK1 knockdown efficiency was significant (Figure 2a). Colony formation assay showed that circTLK1 silencing inhibited the proliferation of Caki-1 and 786-O cells (Figure 2b). In addition, flow cytometry showed that knockdown of circTLK1 induced cell cycle arrest of Caki-1 and 786-O cells in G1 phase and accelerated apoptosis (Figure 2c and d). Scratch and transwell assays revealed that transfection with si-circTLK1 suppressed the migration and invasion of Caki-1 and 786-O cells (Figure 2e–g). Moreover, western blot analysis showed that depletion of circTLK1 led to a marked increase in E-cadherin level and a significant decrease in Vimentin level (Figures 2h and i). Overall, these results indicated that down-regulation of circTLK1 suppressed the proliferation and metastasis of RCC cells and promoted apoptosis.

### 3.3 CBL silencing inhibits proliferation and metastasis and induces apoptosis in RCC cells

**Figure 3:**
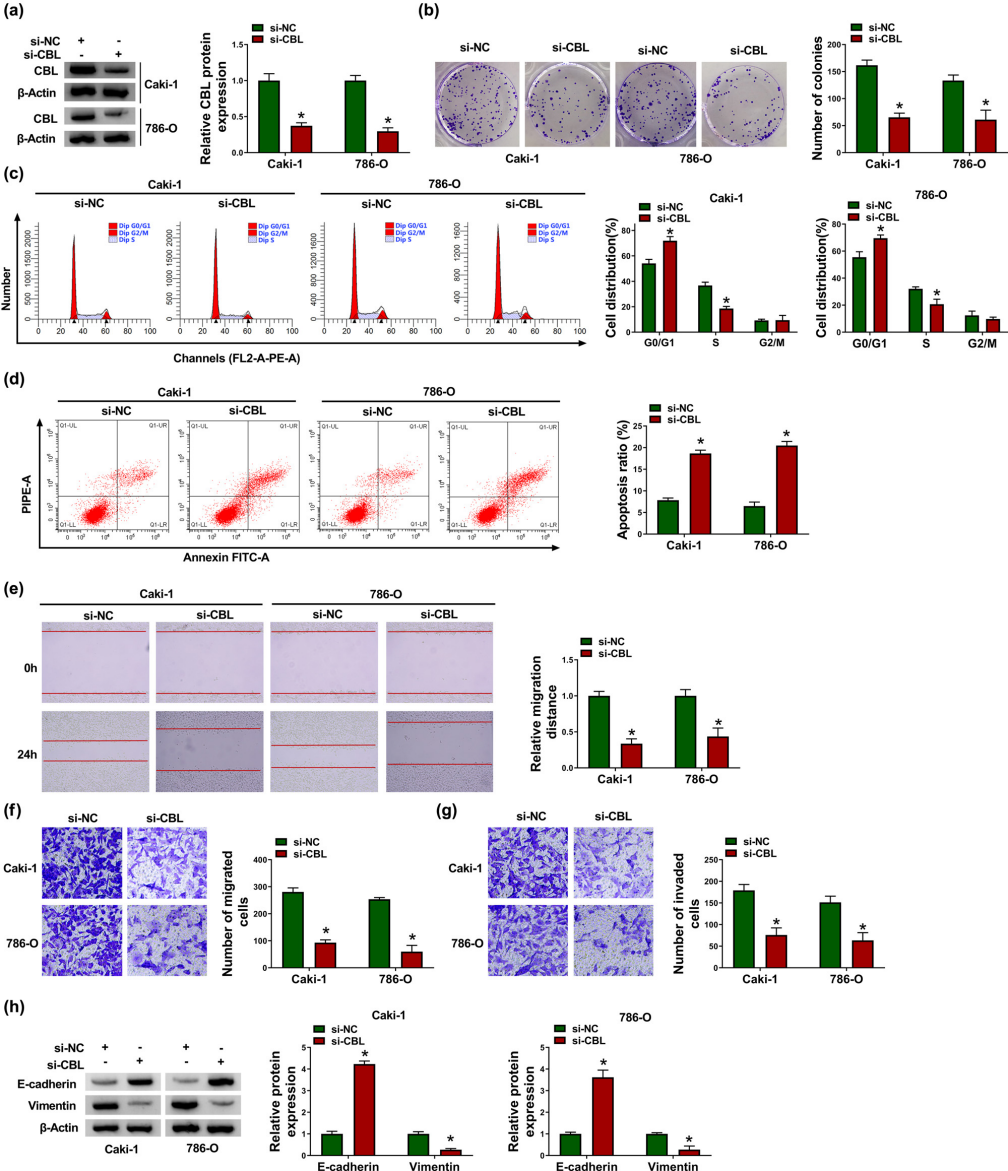
CBL silencing inhibits proliferation and metastasis and induces apoptosis in RCC cells. Caki-1 and 786-O cells were introduced with si-NC or si-CBL. (a) CBL protein level was measured by western blot. (b) Cell proliferation was assessed by colony formation assay. (c and d) Cell distribution and apoptosis ratio were determined by flow cytometry. (e–g) Cell migration and invasion were evaluated by scratch assay and transwell assay. (h) The protein levels of E-cadherin and Vimentin were examined by western blot. *P < 0.05.

Next, a series of functional experiments were performed in Caki-1 and 786-O cells transfected with si-CBL to explore the role of CBL in RCC progression. First of all, the knockdown efficiency of CBL was determined by western blot assay (Figure 3a). Subsequently, colony formation assay suggested that si-CBL transfection significantly reduced the proliferation ability of Caki-1 and 786-O cells (Figure 3b). Flow cytometry illustrated that silencing of CBL expedited cycle arrest and apoptosis of Caki-1 and 786-O cells (Figures 3c and d). In addition, scratch assay exhibited that knockdown of CBL impeded the migration of Caki-1 and 786-O cells (Figure 3e). Furthermore, transwell assay showed that the migration and invasion of Caki-1 and 786-O cells were inhibited after si-CBL transfection (Figure 3f and g). In addition, CBL down-regulation blocked EMT in Caki-1 and 786-O cells by increasing E-cadherin and decreasing Vimentin (Figure 3h). Collectively, these data evidenced that knockdown of CBL impeded RCC cell proliferation and metastasis and induced apoptosis.

### 3.4 CBL overexpression reverses the effect of circTLK1 depletion on RCC cell progression

**Figure 4:**
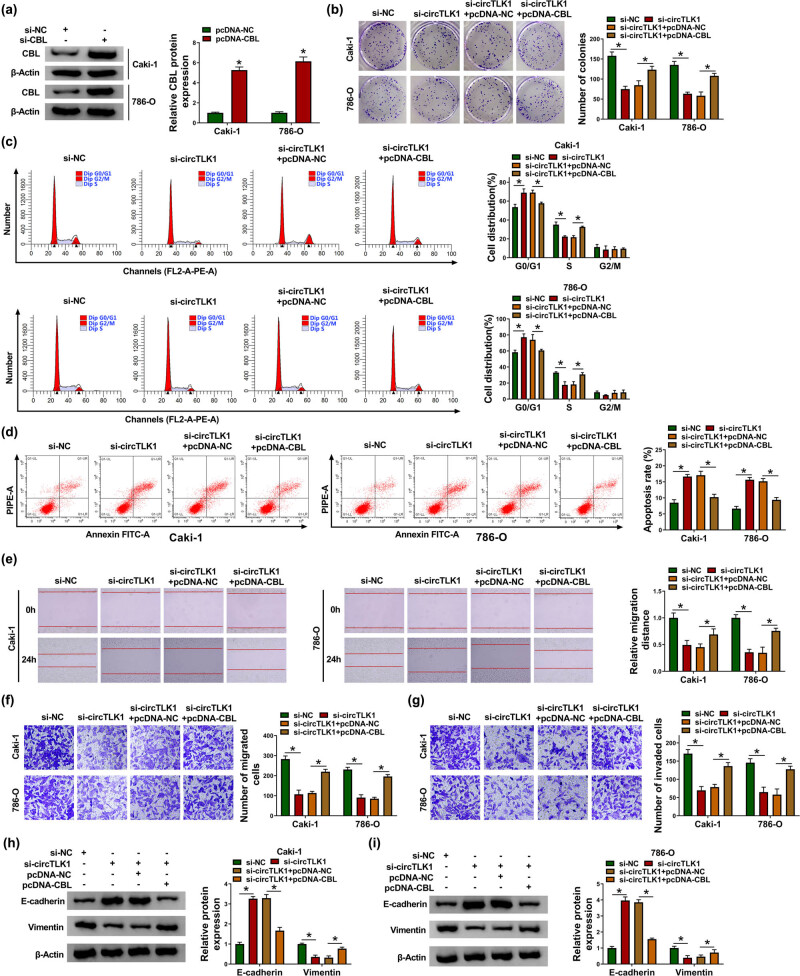
CBL overexpression reverses the effect of circTLK1 depletion on RCC cell progression. (a) The overexpression efficiency of CBL was tested by western blot. After transducing Caki-1 and 786-O cells with si-NC, si-circTLK1, si-circTLK1 + pcDNA-NC, or si-circTLK1 + pcDNA-CBL, colony number (b), cell distribution (c), apoptosis rate (d), cell migration (e and f), cell invasion (g), and the levels of EMT-related proteins (h and i) were detected via appropriate methods. *P < 0.05.

In view of the functions of circTLK1 and CBL in RCC cells, we further explored whether the influence of circTLK1 on RCC cell progression is related to CBL. As depicted in Figure 4a, CBL protein level in Caki-1 and 786-O cells transfected with pcDNA-CBL was significantly up-regulated, indicating successful transfection. Subsequently, si-circTLK1 and pcDNA-CBL were co-transfected into Caki-1 and 786-O cells to investigate their effects on RCC cell progression. The results showed that knockdown of circTLK1 markedly impeded cell proliferation (Figure 4b) and induced cell cycle arrest (Figure 4c) and apoptosis (Figure 4d) in Caki-1 and 786-O cells, while these effects were abolished by up-regulating CBL. In addition, inhibition of circTLK1 remarkably suppressed cell migration (Figure 4e and f), invasion (Figure 4g), and EMT (Figure 4h and i) in Caki-1 and 786-O cells, whereas these impacts were abrogated after transfection with pcDNA-CBL. These data evidenced that circTLK1 affected RCC cell progression by regulating CBL.

### 3.5 circTLK1 directly interacts with miR-495-3p

**Figure 5:**
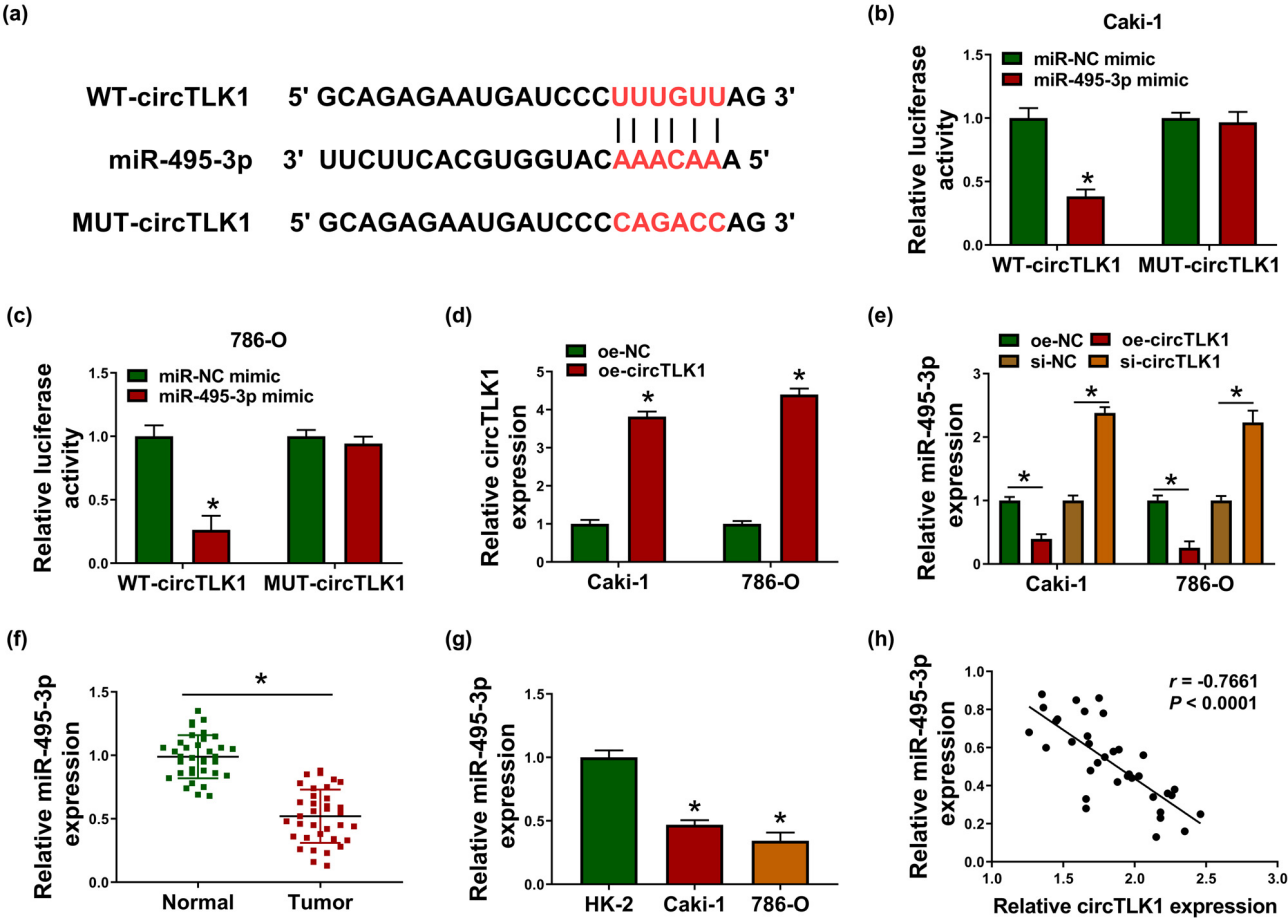
circTLK1 directly interacts with miR-495-3p. (a) Circular RNA Interactome predicted the putative binding site of circTLK1 and miR-495-3p. (b and c) The luciferase activity was tested in Caki-1 and 786-O cells co-transfected with WT-circTLK1 or MUT-circTLK1 and miR-NC mimic or miR-495-3p mimic. (d) circTLK1 expression was detected in Caki-1 and 786-O cells after transfection with oe-NC or oe-circTLK1. (e) The level of miR-495-3p was measured in Caki-1 and 786-O cells transfected with oe-NC, oe-circTLK1, si-NC, or si-circTLK1. (f and g) miR-495-3p expression was examined in RCC tissues and cells. (h) The correlation between circTLK1 and miR-495-3p was analyzed by Spearman’s correlation coefficient. *P < 0.05.

To discover the molecular mechanism of circTLK1 regulating RCC progression, bioinformatics analysis was used to predict miRNAs with a targeting relationship with circTLK1. Circular RNA Interactome online software found that circTLK1 and miR-495-3p have a possible binding site (Figure 5a). Then, dual-luciferase reporter assay suggested that miR-495-3p mimic overtly reduced the luciferase activity of WT-circTLK1 reporter in Caki-1 and 786-O cells (Figure 5b and c). In addition, circTLK1 expression was strikingly increased after transfection with oe-circTLK1 (Figure 5d). Moreover, circTLK1 overexpression drastically restrained miR-495-3p expression, while circTLK1 silencing remarkably promoted miR-495-3p expression (Figure 5e). Compared with normal tissues, miR-495-3p level was significantly decreased in RCC tissues (Figure 5f). Consistently, miR-495-3p level in Caki-1 and 786-O cells was prominently reduced compared to HK-2 cells (Figure 5g). Spearman’s correlation analysis showed that circTLK1 and miR-495-3p were negatively correlated in RCC tissues (Figure 5h). These data indicated that circTLK1 targeted miR-495-3p in RCC.

### 3.6 miR-495-3p directly targets CBL

**Figure 6:**
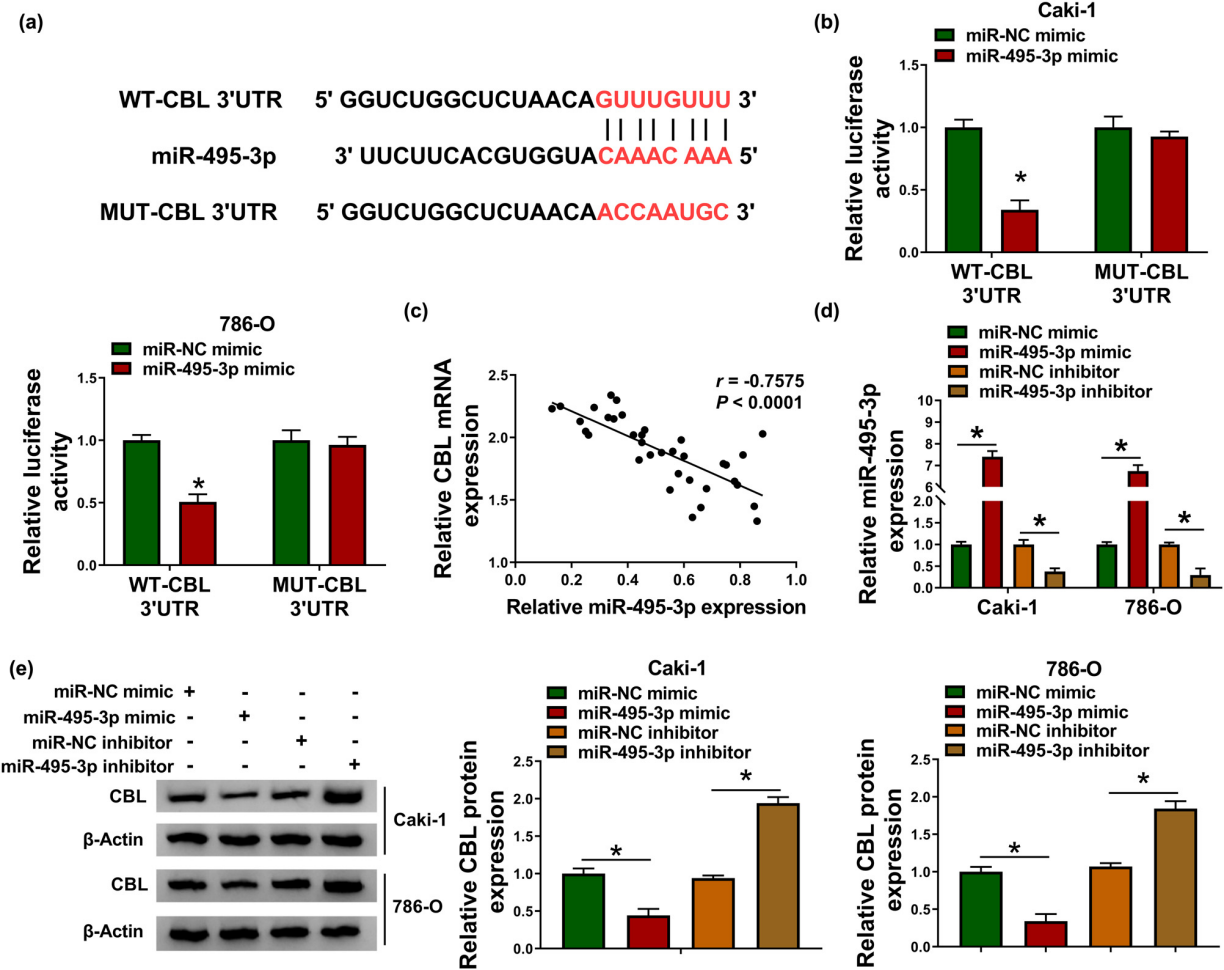
miR-495-3p directly targets CBL. (a) The predicted binding site of miR-495-3p and CBL 3′UTR was displayed. (b) The luciferase activity was detected in Caki-1 and 786-O cells after transfection with WT-CBL 3′UTR or MUT-CBL 3′UTR and miR-NC mimic or miR-495-3p mimic. (c) The correlation between miR-495-3p and CBL in RCC tissues was tested by Spearman’s correlation coefficient. (d and e) The levels of miR-495-3p and CBL protein were examined in Caki-1 and 786-O cells transfected with miR-NC mimic, miR-495-3p mimic, miR-NC inhibitor, or miR-495-3p inhibitor. *P < 0.05.

Next, bioinformatics software TargetScan predicted that miR-495-3p might bind to CBL 3′UTR (Figure 6a). Subsequently, dual-luciferase reporter analysis showed that mature miR-495-3p remarkably decreased the luciferase activity of WT-CBL 3′UTR reporter in Caki-1 and 786-O cells (Figure 6b). Spearman’s correlation coefficient illustrated that miR-495-3p was negatively correlated with CBL in RCC tissues (Figure 6c). Besides, Caki-1 and 786-O cells were introduced with miR-495-3p mimic or miR-495-3p inhibitor, and qRT-PCR analysis showed significant miR-495-3p overexpression and knockdown efficiency (Figure 6d). Furthermore, up-regulation of miR-495-3p markedly inhibited CBL protein expression, whereas down-regulation of miR-495-3p overtly facilitated CBL protein expression (Figure 6e). These data evidenced that CBL was a target of miR-495-3p.

### 3.7 circTLK1 regulates CBL by sponging miR-495-3p

**Figure 7:**
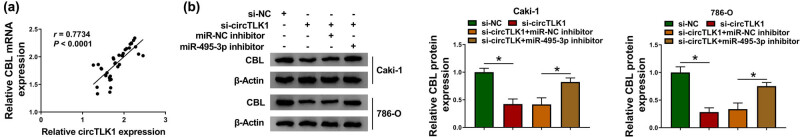
circTLK1 regulates CBL by sponging miR-495-3p. (a) Spearman’s correlation coefficient was used to analyze the correlation between circTLK1 and CBL mRNA in RCC tissues. (b) CBL protein level was measured by western blot in Caki-1 and 786-O cells transfected with si-NC, si-circTLK1, si-circTLK1 + miR-NC inhibitor, or si-circTLK1 + miR-495-3p inhibitor. *P < 0.05.

To elucidate the relationship among circTLK1, miR-495-3p, and CBL in RCC cells, Caki-1 and 786-O cells were transfected with si-NC, si-circTLK1, si-circTLK1 + miR-NC inhibitor, or si-circTLK1 + miR-495-3p inhibitor. In RCC tissues, circTLK1 expression was positively correlated with CBL mRNA expression (Figure 7a). As displayed in Figure 7b, co-transfection of si-circTLK1 and miR-495-3p inhibitor alleviated the reduction in CBL protein level caused by circTLK1 knockdown alone. These results indicated that circTLK1 regulated CBL expression by sponging miR-495-3p.

### 3.8 circTLK1 silencing blocks tumor growth *in vivo*

**Figure 8:**
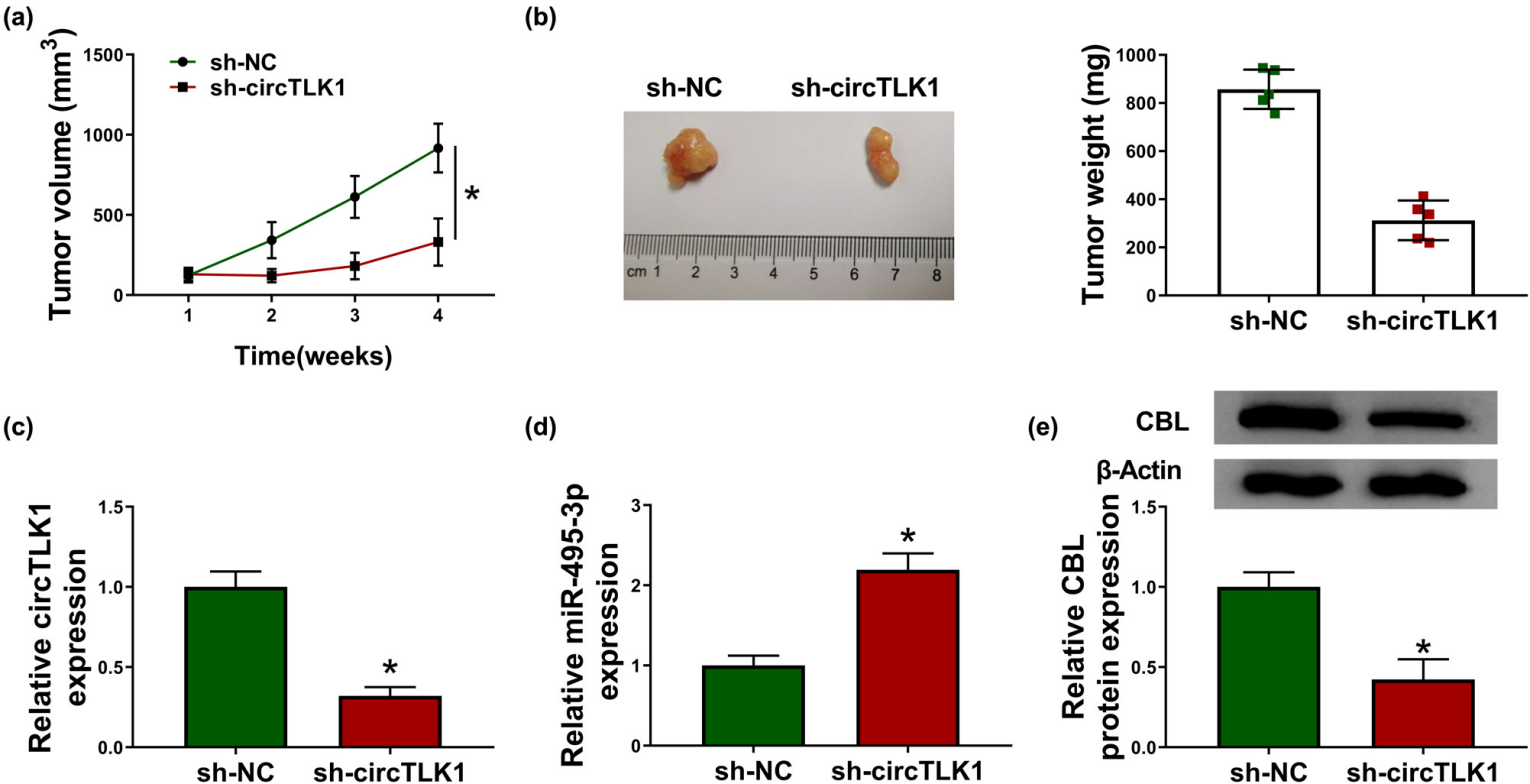
circTLK1 silencing blocks tumor growth *in vivo*. 786-O cells transfected with sh-NC or si-circTLK1 were subcutaneously injected into the nude mice (*n* = 5 per group). (a) Tumor volume was measured once a week. (b) After 4 weeks, the mice were killed, and the xenograft tumors were removed and weighed. (c–e) The levels of circTLK1, miR-495-3p, and CBL in xenograft tumors were detected using qRT-PCR or western blot. *P < 0.05.

To explore the role of circTLK1 in tumorigenesis *in vivo*, we constructed a xenograft mouse model by introducing sh-circTLK1 into 786-O cells. As shown in Figure 8a, tumor volume was remarkably decreased in the sh-circTLK1 group compared to the sh-NC group. Simultaneously, tumor weight in the sh-circTLK1 group was prominently lower than that in the sh-NC group (Figure 8b). In addition, the levels of circTLK1 and CBL protein were strikingly reduced, while miR-495-3p level was significantly increased in the sh-circTLK1 group compared with the sh-NC group (Figure 8c–e). These results indicated that circTLK1 knockdown inhibited tumor growth *in vivo*.

## 4 Discussion

With the rapid development of high-throughput sequencing technology, multiple circRNAs related to tumor progression have been identified [18]. Accumulating evidence has certified that dysregulation of circRNAs is extensively implicated in the development of various diseases, including cancer [19]. Moreover, circRNAs have been confirmed to occupy an important position in the pathogenesis of kidney diseases, including RCC [20]. Therefore, elucidating the molecular mechanism of circRNAs is essential for RCC treatment. In our research, the biological function and potential mechanism of circTLK1 in RCC were investigated in depth. Previous studies revealed that circTLK1 was conspicuously up-regulated in patients with acute ischemic stroke and RCC, which was consistent with the results of this research [12,21]. Besides, loss-of-function experiments indicated that circTLK1 silencing decelerated proliferation and metastasis and facilitated apoptosis in Caki-1 and 786-O cells, which was in agreement with the previous results [12].

Emerging evidence has suggested that circRNAs act as miRNA sponges and regulate biological functions by mediating gene expression through competing endogenous RNA (ceRNA) mechanisms [22]. In this research, we found that circTLK1 might sponge miR-495-3p based on bioinformatics analysis. Investigations have shown that miR-495-3p serves as a tumor-suppressing factor in various cancers, including gastric carcinoma [23], melanoma [24], and prostate cancer [25]. In clear cell RCC, LUCAT1 accelerated cell growth and invasion via targeting miR-495-3p [26]. However, the potential mechanism of miR-495-3p in RCC progression needs further study. In the present research, circTLK1 directly targeted miR-495-3p to regulate CBL expression.

Moreover, increasing evidence has verified that miRNAs weaken gene expression by binding to 3′UTR of mRNAs [27]. This research confirmed that miR-495-3p was directly combined with CBL. CBL is a proto-oncogene encoding E3 ubiquitin ligase [28]. CBL mutations play a crucial role in many cancers, including acute myeloid leukemia [29]. In hepatocellular carcinoma, miR-486-5p overexpression hindered cell proliferation and motility through repressing CBL [30]. In breast cancer, miR-124-3p ameliorated the malignancy of tumor via down-regulating CBL [31]. A previous research unveiled that CBL up-regulation alleviated the inhibition of miR-200a-3p overexpression on RCC progression [32]. In the current research, CBL was also strikingly up-regulated in RCC. Furthermore, this research found that silencing of CBL suppressed cell proliferation and metastasis and triggered apoptosis in RCC.

In conclusion, these findings discovered that circTLK1 contributed to the growth and metastasis of RCC cells by sponging miR-495-3p to indirectly modulate CBL. The discovery of the new ceRNA mechanism of circTLK1/miR-495-3p/CBL might provide a new therapeutic approach for RCC.
